# Annual rhythms of temporal niche partitioning in the Sparidae family are correlated to different environmental variables

**DOI:** 10.1038/s41598-018-37954-0

**Published:** 2019-02-08

**Authors:** Valerio Sbragaglia, Jesús D. Nuñez, Davide Dominoni, Salvatore Coco, Emanuela Fanelli, Ernesto Azzurro, Simone Marini, Marc Nogueras, Massimo Ponti, Joaquin del Rio Fernandez, Jacopo Aguzzi

**Affiliations:** 1 Institute for Environmental Protection and Research (ISPRA), Via del Cedro 38, 57122 Livorno, Italy; 2 Department of Biology and Ecology of Fishes, Leibniz-Institute of Freshwater Ecology and Inland Fisheries, Müggelseedamm 310, Berlin, Germany; 3 IIMyC, Instituto de Investigaciones Marinas y Costeras, CONICET – FCEyN, Universidad Nacional de Mar del Plata, Funes, 3250(7600) Mar del Plata, Provincia de Buenos Aires Argentina; 4 Department of Animal Ecology, Netherlands Institute of Ecology (NIOO-KNAW), P.O Box 50, 6700 AB Wageningen, The Netherlands; 5 Institute of Biodiversity, Animal Health and Comparative Medicine, University of Glasgow, Glasgow, G128QQ UK; 6 Dipartimento di Scienze Biologiche, Geologiche e Ambientali, University of Bologna, Via S. Alberto 163, 48123 Ravenna, Italy; 7 Department of Life and Environmental Sciences, Polytechnic University of Marche, Via Brecce Bianche, 60131 Ancona, Italy; 8 Stazione Zoologica A Dohrn, Villa comunale, Napoli, Italy; 9 Institute of Marine Science, National Research Council of Italy, Forte Santa Teresa, la Spezia, Italy; 10 SARTI Research Group. Dept. Eng. Electrònica, Universitat Politècnica de Catalunya, Vilanova i la Geltrú, Spain; 11 Consorzio Nazionale Interuniversitario per le Scienze del Mare (CoNISMa), Piazzale Flaminio 9, 00196 Roma, Italy; 12 Marine Science Institute (ICM-CSIC), Passeig Marítim de la Barceloneta 37-49, Barcelona, Spain

## Abstract

The seasonal timing of recurring biological processes is essential for organisms living in temperate regions. While ample knowledge of these processes exists for terrestrial environments, seasonal timing in the marine environment is relatively understudied. Here, we characterized the annual rhythm of habitat use in six fish species belonging to the Sparidae family, highlighting the main environmental variables that correlate to such rhythms. The study was conducted at a coastal artificial reef through a cabled observatory system, which allowed gathering underwater time-lapse images every 30 minutes consecutively over 3 years. Rhythms of fish counts had a significant annual periodicity in four out of the six studied species. Species-specific temporal patterns were found, demonstrating a clear annual temporal niche partitioning within the studied family. Temperature was the most important environmental variable correlated with fish counts in the proximity of the artificial reef, while daily photoperiod and salinity were not important. In a scenario of human-induced rapid environmental change, tracking phenological shifts may provide key indications about the effects of climate change at both species and ecosystem level. Our study reinforces the efficacy of underwater cabled video-observatories as a reliable tool for long-term monitoring of phenological events.

## Introduction

In temperate regions, characterized by strong seasonality, the annual temporal organization of biological processes (i.e. phenology) provides evident ecological advantages^1,2^. One of the mechanisms governing annual rhythms implies the existence of an internal time-keeping mechanism (or circannual clock) that is able to synchronize with external cues in order to cope with, and anticipate, predictable changes in the environment^3^. Photoperiod is the most important proximate variable controlling phenology in animals but a certain degree of plasticity allows non-photoperiodic variables to modulate annual timing programs, to cope with unpredictable changes of environment. For example, the duration of the photophase is the principal environmental variable controlling full gonadal maturation in birds, but temperature, rainfall, and food contribute to the fine tuning of the process^4^. The regulation of phenology is not well known in marine organisms, and much less studied, compared to terrestrial ones^5,6^. For example, photoperiod in combination with temperature and food availability seem to have a synergistic effect on the synchronization of annual biological processes in fish^7,8^. Moreover, investigations *in situ* are rarely performed, due to the technical and pragmatic difficulties of surveying the marine environment at high frequency and large temporal scales.

Several authors have already stressed that the ultimate significance of biological rhythms cannot be completely understood by presenting a single variable to individuals maintained in the laboratory, because the observed response may not be normally expressed, or relevant to fitness, as in the wild^3,9,10^. Studying species in their natural ecosystems allows exploring the temporal modulation of biological processes in the presence of photoperiodic and non-photoperiodic signals, as well as under intra- and interspecific competition^11,12^. Accordingly, expanding the study of biological rhythms to natural contexts and to multiple species allows a comprehensive understanding of evolutionary mechanisms of phenology^13^. Such an approach is complementary to more mechanistic ones in the laboratory and it may add new perspectives to track the effects of key environmental drivers such as temperature, photoperiod, and salinity on phenology of marine species. This knowledge is of particular relevance today, to better understand how marine organisms are responding to climate change^14,15^, with cascade effects influencing both community structures and ecosystems’ functioning (for a review see^11^).

In the last two decades, there have been substantial developments in telemetry systems, allowing continuous long-term tracking of aquatic animals^16–18^. At the same time, multiparametric video-platforms cabled to shore for continuous data transmission and powering (i.e. cabled observatories) have become an effective tool to investigate underwater ecosystems^19–22^. Although the spatial area that cabled observatories can monitor is limited, they have the advantage to enable monitoring specific habitats for very long periods of time^23,24^ and in automatic way^22^. In this context, the coastal Seafloor Observatory OBSEA^25^, has already demonstrated its value for the video-monitoring of daily activity rhythms of fishes at an artificial reef located in north western Mediterranean Sea, 4 Km off the coast and at a depth of 21 m^22,26,27^.

Seasonality in the Mediterranean Sea is well marked and the reproductive timing of coastal rocky fishes is correlated with seasonal changes of photoperiod, temperature, and salinity^28^. One of the most abundant taxa in the Mediterranean coastal fish assemblages are those belonging to sea breams, family Sparidae^29,30^. They are also commonly observed at artificial reefs^31^ and have a great commercial value in fishery^32^. Despite the fact that some of the species belonging to the Sparidae family have been observed to use artificial reefs differently throughout the year^31^, knowledge about the environmental variables that affect annual changes in species presence is limited^31^. Here, we aimed to identify how water temperature, daily photoperiod, and salinity correlate with the annual habitat use of an artificial reef by six species belonging to the Sparidae family by using 30-minute time-lapse images shot at the OBSEA cabled observatory over a three year period. The artificial reef is located in north western Mediterranean Sea, 4 Km off the coast and at a depth of 21 m. The six selected species were: the common dentex, *Dentex dentex* (Linnaeus, 1758), the white seabream, *Diplodus sargus* (Linnaeus, 1758), the two-banded seabream, *Diplodus vulgaris* (Geoffroy Saint-Hilaire, 1817), the annular seabream, *Diplodus annularis* (Linnaeus, 1758), the sharpsnout seabream, *Diplodus puntazzo* (Walbaum, 1792), and the zebra seabream, *Diplodus cervinus* (Lowe, 1838).

## Materials and Methods

### Data collection

**Figure 1:**
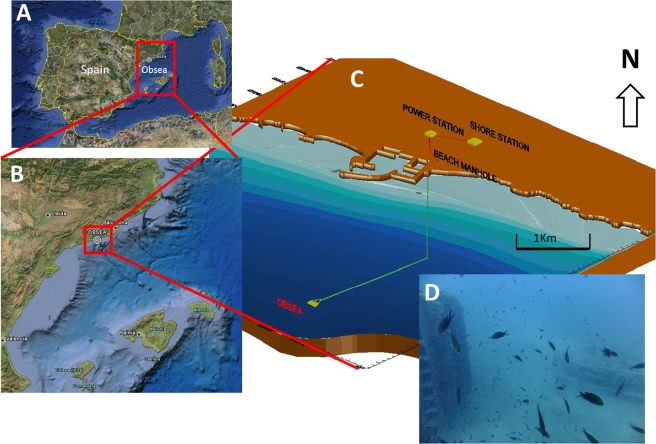
Location of the OBSEA platform in the North Western Mediterranean Sea (**A**,**B**,**C**) Indicates the location of the platform off the harbor of Vilanova i la Geltrú (Spain). (**D**) Depicts the view of the platform (right) and part of the artificial reef (left). Satellite images (**A**,**B**) have been obtained on google maps (©2018 Google, Inst. Geogr., last accessed on 16 August 2018).

The Western Mediterranean Expandable SEAfloor Observatory (OBSEA; www.obsea.es) is a cabled video-platform located at a depth of 21 m, 4 km off Vilanova i la Geltrú, Spain (41°10′54″ N; 01°45′08″ E, geodetic datum WGS84; see Fig. 1A). The observatory is equipped with an underwater camera (OceanOptic Cam) that can store online all acquired time-lapse images. The OBSEA is also equipped with a custom developed LED lighting system to allow shooting at night. There are two light sources located beside the camera at 1 m distance from each other. Each source has one LED emitting 2900 lumen with an angle of 120°. The light has a color temperature of 2700 kelvin (for more details see^25^). The OBSEA is placed in front of an artificial reef at a distance of 3.5 m.

We acquired images every 30 min during 3 years (2012–2014), preserving the same field of view centered on the artificial reef (Fig. 1D). Shooting at night was carried out by switching on the lighting system just before the shooting of the camera, and switching off immediately after (total time of light-on was at about 30 s; for more details see^27^). Temperature and salinity were measured by the CTD probe installed aside the camera. Global irradiance (W m^−2^) has been retrieved from a nearby meteorological station at Sant Pere de Ribes, 6 km away from the OBSEA (http://www.obsea.es).

### Statistical analysis

The acquired images were manually analyzed by a trained operator to count all the individuals belonging to the 6 target species. The final matrix of daily abundances and averaged environmental data was used for the statistical analysis and it was composed by: one dependent variable (daily sum of fish counts); three fixed effects (mean daily water temperature, mean daily salinity, and daily hours of light received at the meteorological station). We investigated which environmental variable was related the most with the changes in fish counts as a proxy of annual rhythms in their habitat use, through Generalized Additive Models for Location, Scale, and Shape (GAMLSS). GAMLSS offers the possibility to select the distribution for the response variable from a very general family of distributions, including highly skewed or kurtotic continuous and discrete distributions. This fits well with the distribution of our dependent variable that is a count variable^33^. We looked at the frequency distribution of the count data of each of the 6 species (Fig. S1), then we used a null model to test three preselected family distributions (Negative Binomial, NBI; Zero-Inflated Poisson, ZIP; Poisson distribution, PO). Afterwards, we used the results of the model to assess the best fitting distribution comparing the Akaike’s Information Criterion (AIC)^34^ and examining normality of residuals by plotting theoretical quantiles *versus* standardized residuals (Q–Q plots) (Table S1). According to the results, we decided to use the NBI family distribution for all the species. The potential temporal autocorrelation was incorporated using Generalized Auto-Regressive Moving Average models (GARMA) with “garmaFit” command in “gamlss.utils” library^33^. We used a null model to test different combination of GARMA structure (See Table S1). Then, we used the results of the model to assess the best autocorrelation structure using AIC and examining the presence of autoregressive conditional heteroscedasticity by examining Auto-Correlation Function plots (ACF) of squared residuals from the models (Fig S2). According to the results, we decided the best autocorrelative structure for each species-specific model (Table S2).

Since the number of photos counted for each day was different due to technical difficulties (e.g. transient turbidity or camera malfunctioning, see Table S3), we used the logarithm of number of photo counted for each day as an offset variable in the model equation. Prior to analysis, multicollinearity among predictors in the GAMLSS models was tested, using Variance Inflation Factor (VIF). The estimated VIF showed low levels of collinearity among predictors (VIF < 5 for all the cases). Thus, all the predictors were included in the GAMLSS models. We first tested whether the 3-years’ fish count time series indicated an annual periodicity, by implementing a null model with presence/absence of the independent variable periodicity (a vector from 1 to 12 representing the months of the year). In a second step, we implemented all the possible models (n = 8) combining presence/absence of the fixed effects except periodicity (Table S4). In both cases, the AIC was used to assess models’ performance. In addition, we computed Akaike’s weight (w_i_) for each candidate model (Franklin *et al*., 2001) through its computed AIC and the Δ values. The weights’ range between 0 and 1 has been interpreted as the weights of evidence in favor of model *i* as the best model among the set of all candidate models examined^35^. Finally, the models with the smallest AIC and the higher w_i_ values were chosen as the models that best represented the data. In cases where the top models had close convergence (models that did not exceed 0.5 of AIC weight) we implemented a model averaging process to calculate the Relative Importance (RI) of the explicative variables. For this, we used the models that constitute a cumulative AIC weight of 0.95^36^. We considered an RI around 0.9 as a strong explanatory variable, around 0.9–0.6 as moderate and less than 0.6 as weak^36^. All analyses were conducted in R 3.3.1^37^.

## Results

The number of recorded images during the 3-year sampling was 52,609, while those used for species counting were 40,989 (78%). The other photos were of poor quality and were discarded (see Table S3). The total counts for all the targeted species was 125,239: *D. vulgaris* (89,767); *D. annularis* (20,577); *D. sargus* (11,420); *D. dentex* (1,359); *D. cervinus* (1,119); *D. puntazzo* (997). These 6 species together represented 58% of the total fish counts recorded (data for other species not shown).

**Figure 2:**
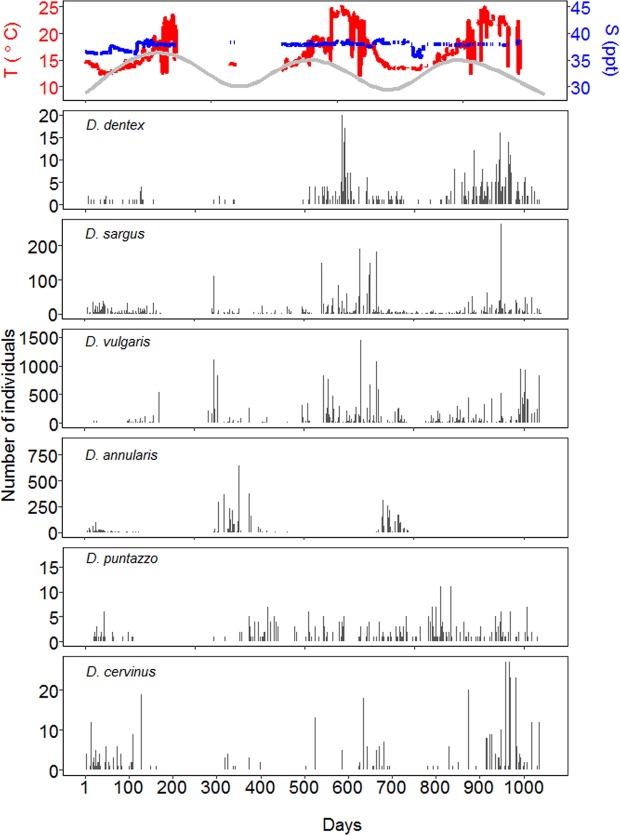
Time series of the daily environmental conditions and the number of individuals counted at the artificial reef according to the days of the three-years study presented here. The plot at the top provides the information on temperature of the water (red), salinity (blue) and daily photoperiod, which is not scaled but ranges from 9 to 16.5 hours (grey line). The bars in the other plots represent the daily counts (number of individuals counted) of each of the six species at the artificial reef.

Modelling results on rhythmicity revealed significant annual oscillations in four out of the six species studied (Table 1) and peaks of fish counts were registered between August and December (Figs 2 and S3). Specifically, *D. dentex* counts reached their maximum in August; *D. vulgaris* and *D. sargus* counts peaked in October and *D. annularis* in December (Figs 2 and S3). Differently, the abundance of *D. cervinus* and *D. puntazzo* did not show clear annual rhythmicity (Figs 2 and S3).

**Table 1:** The results of the models used to test periodicity of fish counts in each of the six species.

Species	Df	Period	AIC_i_	∆_i_	*w*_*i*_
*D. dentex*	**9**	**+**	**1719.1**	**0.0**	**0.982**
	8	—	1727.1	8.0	0.018
*D. vulgaris*	**5**	**+**	**5022.1**	**0.0**	**1**
	4	—	5104.0	82.0	<0.001
*D. sargus*	**5**	**+**	**3469.4**	**0.0**	**1**
	4	—	3500.2	30.8	<0.001
*D. annularis*	**7**	**+**	**1408.8**	**0.0**	**0.8**
	6	—	1411.6	2.8	0.2
*D. puntazzo*	**7**	—	**1803.1**	**0.0**	**1**
	8	+	1850.3	47.2	<0.001
*D. cervinus*	**5**	—	**1307.2**	**0.0**	**0.8**
	6	+	1310.0	2.8	0.2

Df indicates degrees of freedom. +/− indicates the presence/absence of the smoothing effect of annual periodicity. The Akaike’s Information Criterion (AIC_i_), the Akaike’s weight (w_i_) and the Δ_i_ values are reported to show the selection information criteria. The models used are highlighted in bold.

**Figure 3:**
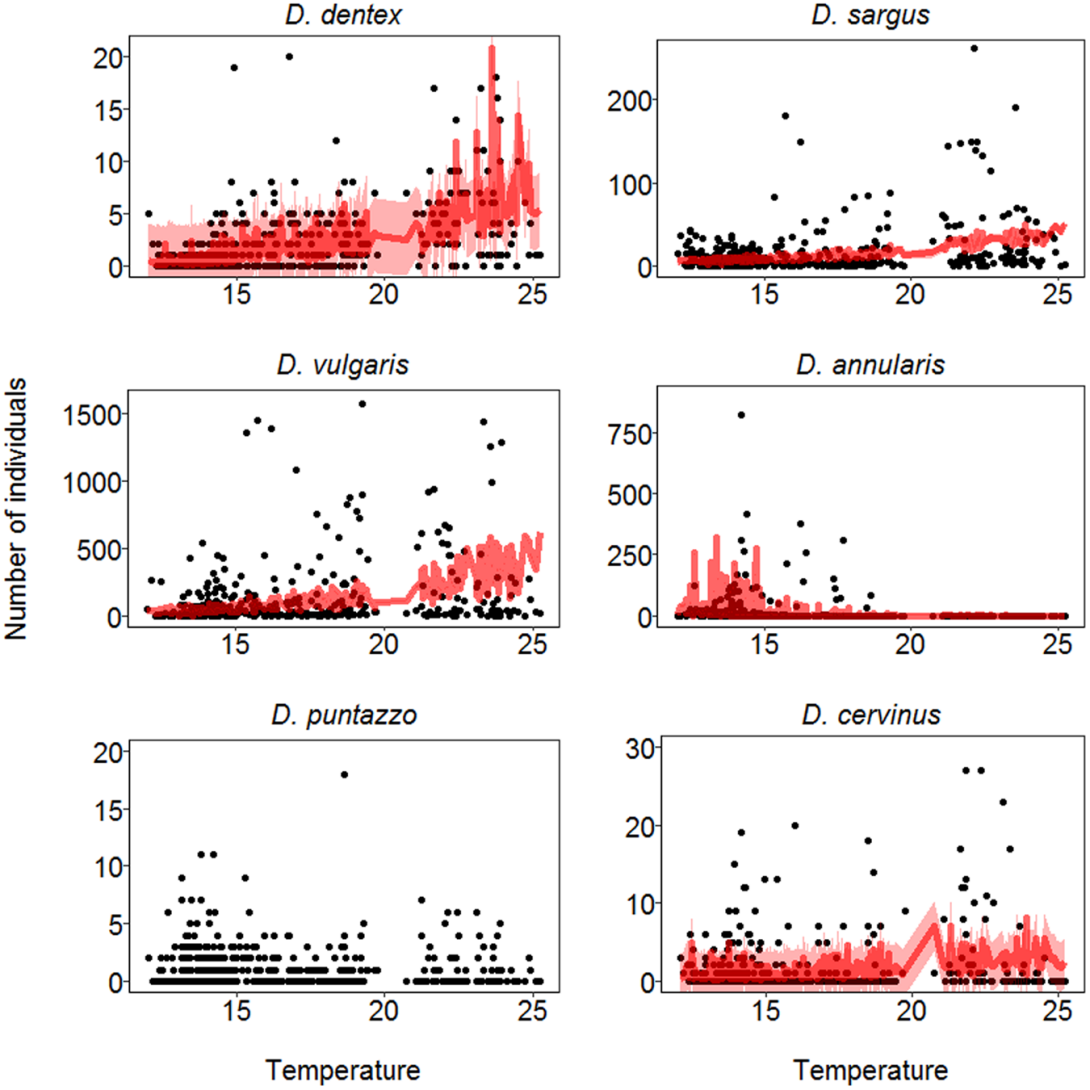
The output of the modeling results according to the best fitting model in Table 2. The points represent the daily counts (number of individuals counted) of the six species at the artificial reef. Each species is represented in relation to daily average of water temperature. The red line represents median prediction (which equals the mean for Negative Binomial I family distribution) together with the 95% confidence interval (red shadowed area).

**Figure 4:**
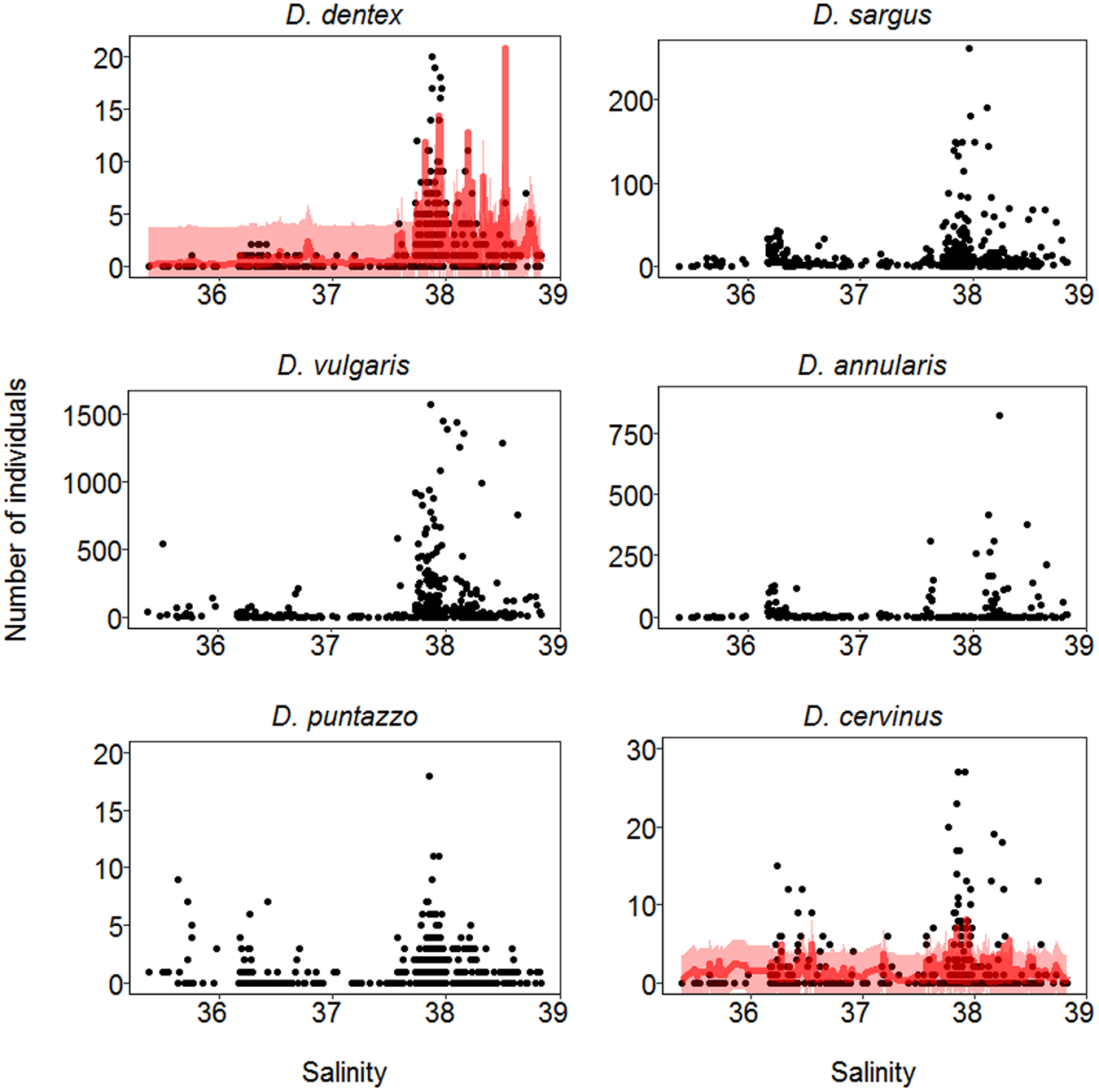
The output of the modeling results according to the best fitting model in Table 2. The points represent the daily counts (number of individuals counted) of the six species at the artificial reef. Each species is represented in relation to daily average of water salinity. The red line represents median prediction (which equals the mean for Negative Binomial I family distribution) together with the 95% confidence interval (red shadowed area).

**Figure 5:**
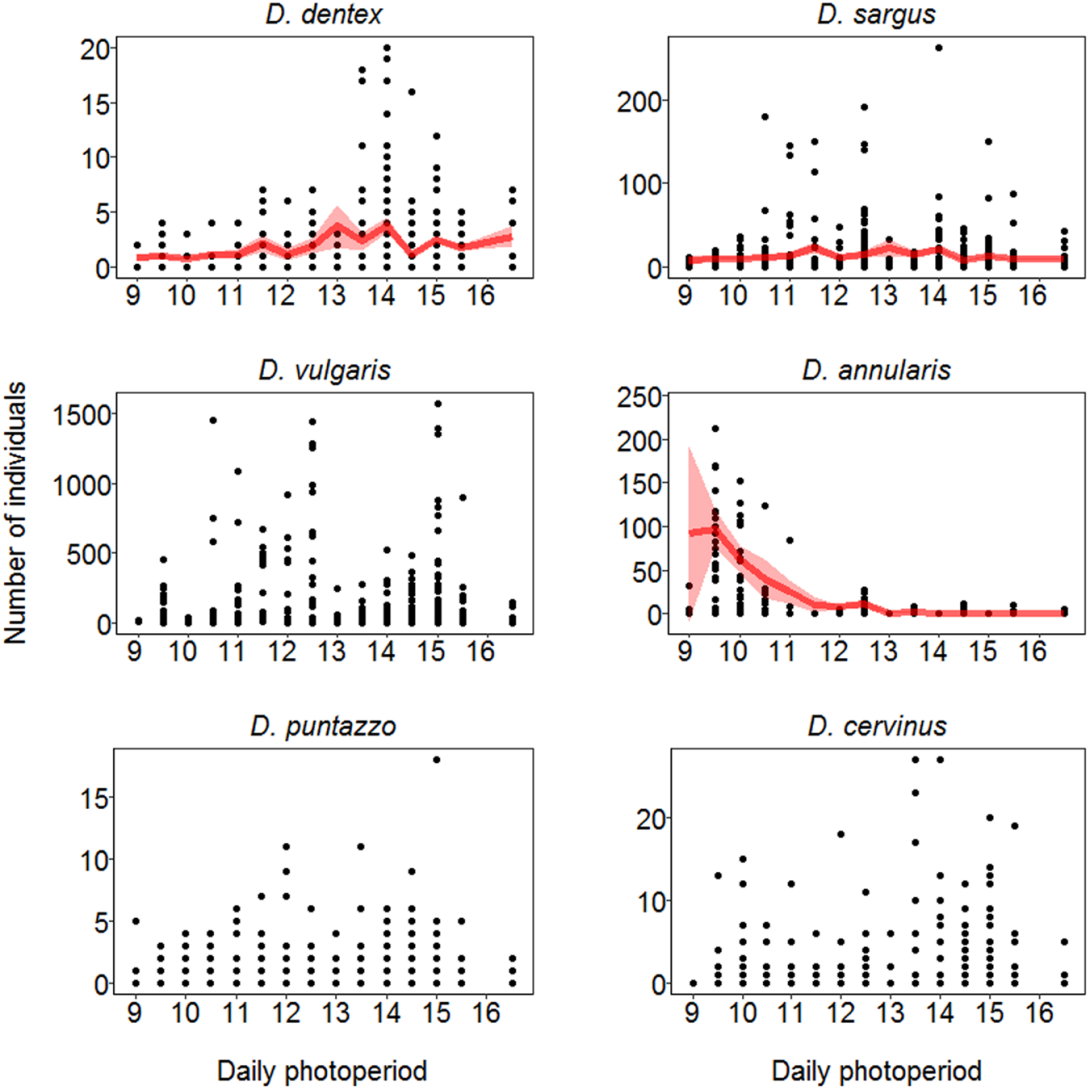
The output of the modeling results according to the best fitting model in Table 2. The points represent the daily counts (number of individuals counted) of the six species at the artificial reef. Each species is represented in relation to the daily photoperiod. The red line represents median prediction (which equals the mean for Negative Binomial I family distribution) together with the 95% confidence interval (red shadowed area).

Modelling results indicated that temporal variations in fish counts were related with different variables, except for *D. puntazzo* counts where none of the fixed effects tested were important (See Table 2). According to the w_i_ and AIC_i_ values, the model that best described counts of *D. dentex* was the one incorporating all the smoothing factors: temperature (Fig. 3 and Table 2), salinity (Fig. 4 and Table 2), and daily photoperiod (Fig. 5 and Table 2). Counts of *D. dentex* started to increase when the water temperature was above 20 °C, with a clear peak when the salinity was at about 38 ppt and daily photoperiod was at about 14 hours (August; see Fig. S3). In the case of *D. sargus*, temperature was the most important smoothing factor (Fig. 3 and Table 2), but the daily photoperiod also indicated a weak (RI = 0.67) smoothing effect (Fig. 5 and Table 2). Counts of *D. sargus* steadily increased from 19 to 25 °C. The model that best described counts of *D. vulgaris* incorporated only temperature as smoothing factor (Fig. 3 and Table 2) and counts of this species indicated a similar pattern to the one of *D. dentex*. Then, counts of *D. annularis* indicated a significant smoothing effect of temperature (Fig. 3 and Table 2) and daily photoperiod (Fig. 5 and Table 2). The presence of the species at the artificial reef was higher at temperatures below 16 °C and with daily photoperiod around 10 hours (December). Finally, the counts of *D. cervinus* indicated a significant smoothing effect of temperature (Fig. 3 and Table 2) with a similar pattern to the one showed by *D. sargus* and a weak effect of salinity (Fig. 4 and Table 2).

**Table 2:** The results of the models used to test the effect of temperature, salinity and daily photoperiod on the fish counts.

Species	Model	Df	Temperature	Salinity	Photoperiod	AIC_i_	∆_i_	*w*_*i*_	Rsq
*D. dentex*	**m1**	**11**	**+**	**+**	**+**	**1627.1**	**0.0**	**0.99**	**0.33**
	m3	10	+	+	—	1637.7	10.6	<0.01	0.29
	m2	10	+	—	+	1640.7	13.6	<0.01	0.32
	m6	10	—	+	+	1641.4	14.1	<0.01	0.20
*D. sargus*	m1	7	+	+	+	3429.5	0.0	0.36	0.19
	m4	6	+	—	+	3430.5	0.3	0.31	0.19
	m3	6	+	+	—	3431.9	1.0	0.22	0.18
	m7	5	+	—	—	3430.5	2.4	0.11	0.18
RI			**0.90**	<0.6	**0.67**				
*D. vulgaris*	m5	5	+	—	—	5037.9	0.0	0.40	0.21
	m3	6	+	+	—	5038.4	0.4	0.32	0.20
	m4	6	+	—	+	5039.8	1.9	0.16	0.20
	m1	7	+	+	+	5040.3	2.3	0.12	0.20
RI			**0.99**	<0.6	<0.6				
*D. annularis*	**m4**	**9**	**+**	—	**+**	**1644.2**	**0.0**	**0.64**	**0.33**
	m1	10	+	+	+	1645.4	1.2	0.36	0.32
	m5	8	+	—	—	1679.0	34.8	<0.01	0.14
	m3	9	+	+	—	1680.7	36.6	<0.01	0.27
*D. puntazzo*	**m8**	**6**	—	—	—	**1411.2**	**0.0**	**0.64**	—
	m6	7	—	—	+	1412.4	1.3	0.34	0.05
	m7	7	—	+	—	1420.4	9.2	<0.01	0.03
	m4	8	+	—	+	1420.9	9.7	<0.01	0.03
*D. cervinus*	**m3**	**7**	**+**	**+**	—	**1303.7**	**0.0**	**0.63**	**0.04**
	m1	8	+	+	+	1305.2	1.7	0.27	0.07
	m8	5	—	—	—	1308.9	5.4	0.04	—
	m7	6	+	—	—	1309.6	6.1	0.03	0.03

The best four out of eight (see Table S4) models are reported. Df represents the degrees of freedom. +/— indicates the presence/absence of the smoothing effect of the variables. The Akaike’s Information Criterion (AIC_i_), the Akaike’s weight (w_i_) and the Δ_i_ values are reported to show the selection information criteria. Rsq represents the pseudo R-squared, while RI represents the Relative Importance of the explicative variables. In cases where the top models had close convergence (w_i_ < 0.5) we implemented a model averaging process to calculate the RI of the explicative variables. Strong or moderate influences of explanatory variables are highlighted in bold.

## Discussion

Here, we characterized the annual rhythms of habitat use in six fish species belonging to the Sparidae family at an artificial reef in the Western Mediterranean Sea by using fish counts at an underwater cabled observatory throughout three years. Fish counts were used as a proxy of habitat used indicating that only four out of the six studied species had significant annual rhythms at the artificial reef. Moreover temporal variations were correlated with different environmental variables, with temperature showing stronger relation with fish counts than salinity and daily photoperiod. Results for each species were discussed comparing our method with telemetry studies and other methodological approaches previously used. Moreover, we interpreted the annual patterns in the context of trophic ecology and reproductive timing for each species. Finally, we highlighted the importance of our results for long-term monitoring of phenology in the context of climate change.

Counts of the common dentex (*D. dentex*) were significantly correlated to water temperature, salinity, and daily photoperiod with a clear peak in August. This observed pattern is also supported by recent telemetry studies in the NW Mediterranean Sea (at about 200 km north respect to the location of the OBSEA), which highlighted how this species has a clear preference for the suprathermoclinal warm water over the colder layer below the thermocline^38^. The common dentex is a predator occupying a high trophic level in coastal trophic niches, and mainly feeds on other coastal fishes and cephalopods^39–41^. The observed peak around August could be related to food availability around the artificial reef or to water temperature that increases metabolic rate and swimming performance of *D. dentex*^42^, and so the probability of being detected by the video monitoring. Finally, our results suggest that the artificial reef is not an important habitat for the spawning of the species that usually occur between March-June^41^.

Counts of the white sea bream (*D. sargus*) were mainly correlated to water temperature and peaked in October. Interestingly, during a telemetry study Aspillaga, *et al*.^43^ observed *D. sargus* performing movements to deeper areas (>20 m) between November-December and March-April. Such behavior was related to the avoidance of stormy conditions in shallow water (November-December), or to reproductive spawning aggregation (between March-May^44^). Our results suggest that the species’ counts decrease at the artificial reef (probably due to displacements of individuals to deeper spawning grounds) between March and May. Interestingly, the average ordinary home-range for this species is less than one square km^43,45,46^ and it could be even smaller in the presence of an artificial reef^47^. Those structures have been demonstrated to be important feeding sites for the omnivorous *D. sargus*^48^, which mainly feed on bivalves, echinoderms, and algae^29^. Thus, the artificial reef is likely used as a foraging ground, supporting individuals’ dietary requirements, just before the onset of their spawning period.

The counts of the two-banded seabream (*D. vulgaris*) showed a similar annual pattern to the one of *D. sargus* (i.e. a peak around October), and they were also significantly correlated to water temperature. Preliminary telemetry study on the *D. vulgaris* did not show extraordinary movements related to the spawning season^49^ with average ordinary home range within one square Km^46^. However, spawning aggregation is reported by other authors^50^. This species is also omnivorous, preferentially feeding on bivalves, crustaceans, polychaetes, echinoderms, and algae^29^. Interestingly, crustaceans are mostly consumed during autumn, while echinoderms are less consumed during summer^51^. Such annual dietary patterns could be the reason of the marked annual rhythm at the artificial reef. Spawning of *D. vulgaris* occurs between December and January, thus the high consumption on crustaceans and the increase of counts at the artificial reef in autumn could be related to the dietary requirements for reproduction. Crustaceans have indeed a high caloric content^52,53^ and a marked preference for crustaceans prey just before the reproductive period has been observed in other sparids, such as *Pagellus erythrinus* (Linnaeus, 1758) and *P. acarne* (Risso, 1827)^54^.

The annular seabream (*D. annularis*) is the only species among the six studied here that showed a peak of annual presence at the artificial reef in December. Such peak was related to water temperature and daily photoperiod. The habitat of *D. annularis* is mostly related to *Posidonia oceanica* meadows^55^ that are abundant around the OBSEA. Individuals probably spend the greater part of the year there, except in winter when they are more present at the artificial reef. Telemetry and tag-recapture experiments showed that the annular seabream has a high site fidelity^56^, and its diet is based on polychaetes, crustaceans, and algae. Interestingly, artificial reefs have been demonstrated to affect *D. annularis* diet by providing greater amounts of copepods^57,58^. It is therefore plausible that the peak of presence at the artificial reef around December is related again to dietary needs, in order to secure energy for the spawning that occurs from February to July^59^.

The sharpsnout seabream’s (*D. puntazzo*) presence is constant at the artificial reef with no significant annual variations in counts. This species is known to preferentially feed at deeper waters than other sparids such as *D. sargus* and *D. vulgaris*^29^. Moreover, the diet of *D. puntazzo* is mainly composed of algae and sponges, while teleosts, mollusks, crustaceans, and annelids represent accessory items of its trophic niche^29,60^. Such differences ecologically segregate *D. puntazzo* from the other most common sparid species such as *D. sargus* and *D. vulgaris*^29^ and indicate a different use of the artificial reef. Telemetry could help in elucidating these aspects, but there are no existing tracking studies on this species to the best of our knowledge. Also, *D. cervinus* did not show significant fluctuations in counts at the artificial reef, although we detected weak correlations of temperature and salinity. Information is scarce on the ecology and biology of this species, however visual census studies reported that *D. cervinus* is more abundant around natural reefs than artificial ones^61^.

It is important to notice that we are unable to control the potential effect of artificial light at night on the counts of the six fish species at the artificial reef. The OBSEA mounts a lighting system that allows filming at night, which was used here intermittently every 30 min for about 30 s (see materials and methods). It is unlikely that short intermittent light emissions at night changed the behavior of fish so much as to affect their annual rhythms of habitat use at the artificial reef. However, future long-term monitoring studies need to take into consideration the potential direct and indirect effects of artificial lights on fish behavior in coastal areas^62^.

Moreover, some of the species belonging to the Sparidae family (*D. sargus*, *D. vulgaris* and *D. puntazzo*) are known to perform ontogenetic shifts in habitat use^63^ that could have biased the annual patterns described here. For example *D.sargus* showed peaks of recruitment (6–7 cm) in October-November coinciding with the peaks of counts at the OBSEA. We were not able to discriminate among recruits, sub-adults and adults size classes in the counted individuals at the OBSEA. So, we cannot exclude that the annual patterns observed here can be partially biased by annual recruitment events.

Our findings show the potential of underwater cabled video-observatories to produce complementary data to commonly employed approaches, such as visual census, stomach content analysis^29^, and telemetry^38^. Our monitoring by a cabled observatory allowed tracking fish assemblages at high frequencies and in the long term, opening new grounds for investigating biological rhythms at the population level but also to monitor and manage coastal ecosystems^22,64^. Despite the fact that cabled observatories are limited in space, the main advantage they offer with respect to a visual census approach is a long-term and low-invasive technology coupled with recent potentiality for automatic counting of individuals^22^ and absolute density estimation^65^. Low invasive sampling approach is particularly important in areas where fishing is allowed; in fact fish behavior can be strongly modulated by fishing pressure^66^. In particular, fish escape response could be negatively modulated by the presence of both SCUBA divers^67^ and free-divers^68^. So, the use of cabled observatory may reveal patterns of presence that could be biased during visual census. However, further research is needed on this topic for a quantitative assessment of other potential bias.

In a world subjected to rapid environmental changes, the huge amount of data generated by video-based tracking technologies provides new opportunities to quantify changes in phenology, which are particularly sensitive indicators of climate change^69^. Temperate coastal marine environments may be particularly vulnerable to changes in water temperature. Here, we showed that annual rhythms of habitat use in the Sparidae family had similar patterns to those observed for reproductive timing and other annual migrations previously described with other methods. Moreover fish counts were mainly (but not only) correlated to water temperature changes. Most importantly, the level of response might significantly differ between closely related species supporting the idea that the impact of climate change within an ecological community is expected to be species-specific, leading to potential phenological mismatches between species occupying different trophic levels and eventually to ecosystem-level impacts^70^.

## Supplementary information

Supplementary info

Original Data

## Data Availability

Original data are available as supplementary file.
