# Supplementary material for: Annual rhythms of temporal niche partitioning in the Sparidae family are correlated to different environmental variables
Source: Sci Rep. 2019 Feb 8;9:1708. doi: 10.1038/s41598-018-37954-0 (PMC6368640; doi:10.1038/s41598-018-37954-0)

Annual rhythms of temporal niche partitioning in the Sparidae family are correlated to different environmental variables

Valerio Sbragaglia[1,2,*], Jesús D. Nuñez[3], Davide Dominoni[4,5], Salvatore Coco[6], Emanuela Fanelli[7], Ernesto Azzurro[1, 8], Simone Marini[9], Marc Nogueras[10], Massimo Ponti[6,11], Joaquin del Rio Fernandez [10], Jacopo Aguzzi[12]

[1] Institute for Environmental Protection and Research (ISPRA), Via del Cedro 38, 57122 Livorno, Italy

[2] Department of Biology and Ecology of Fishes, Leibniz-Institute of Freshwater Ecology and Inland Fisheries, Müggelseedamm 310, Berlin (Germany)

[3] IIMyC, Instituto de Investigaciones Marinas y Costeras, CONICET – FCEyN, Universidad Nacional de Mar del Plata, Funes 3250(7600) Mar del Plata, Provincia de Buenos Aires, Argentina.

[4] Department of Animal Ecology, Netherlands Institute of Ecology (NIOO-KNAW), P.O Box 50, 6700 AB Wageningen, The Netherlands

[5] Institute of Biodiversity, Animal Health and Comparative Medicine, University of Glasgow, Glasgow G128QQ, UK

[6] Dipartimento di Scienze Biologiche, Geologiche e Ambientali, University of Bologna, Via S. Alberto 163, 48123 Ravenna (Italy)

[7] Department of Life and Environmental Sciences, Polytechnic University of Marche, Via Brecce Bianche 60131 Ancona (Italy)

[8] Stazione Zoologica A Dohrn, Villa comunale, Napoli, Italy

[9] Institute of Marine Science, National Research Council of Italy, Forte Santa Teresa, la Spezia.

[10] SARTI Research Group. Dept. Eng. Electrònica. Universitat Politècnica de Catalunya, Vilanova i la Geltrú (Spain)

[11] Consorzio Nazionale Interuniversitario per le Scienze del Mare (CoNISMa), Piazzale Flaminio 9, 00196 Roma, Italy.

[12] Marine Science Institute (ICM-CSIC), Passeig Marítim de la Barceloneta 37-49, Barcelona (Spain)

[*] Corresponding author: valeriosbra@gmail.com

SUPPLEMENTARY MATERIAL

Table S1. Results of the different family distribution tested with the null model for each of the six species. Among the fitting indictors Akaike's Information Criterion (AIC) is in bold because it is the indicator used to select the best family distribution that in all the cases was the (Negative Binomial type I) NBI as indicated by the lower levels of AIC. The other abbreviations stay for: Zero-Inflated Poisson family distribution (ZIP), Poisson family distribution (PO) and Schwarz Bayesian criterion (SBC).

Species	Fitting indicator	Family distribution		
		NBI	ZIP	PO
D. dentex	Global deviance	1782.544	2069.538	2652.031
	**AIC**	**1786.544**	2073.538	2654.031
	SBC	1795.051	2082.046	2658.285
D. sargus	Global deviance	3529.846	13322.19	15480.94
	**AIC**	**3533.846**	13326.19	15482.94
	SBC	3542.354	13334.7	15487.19
D. cervinus	Global deviance	1310.816	1727.001	2830.907
	**AIC**	**1314.816**	1731.001	2832.907
	SBC	1323.324	1739.509	2837.161
D. annularis	Global deviance	1825.257	16959.41	32370.04
	**AIC**	**1829.257**	16963.41	32372.04
	SBC	1837.765	16971.91	32376.3
D. vulgaris	Global deviance	5145.064	125506.3	135874.5
	**AIC**	**5149.064**	125510.3	135876.5
	SBC	5157.571	125518.9	135880.7
D. puntazzo	Global deviance	1422.865	1518.226	1748.583
	**AIC**	**1426.865**	1522.226	1750.583
	SBC	1435.373	1530.733	1754.837

Table S2. Results of the different temporal autocorrelation structure for each of the six species. Among the fitting indictors Akaike's Information Criterion (AIC**)** is in bold because it is the indicator used to select the best temporal autocorrelation structure. SBC stays for Schwarz Bayesian criterion, the first number in brackets corresponds to p (autoregressive order) and the second to q (the moving average order) value specifying the ARMA structure.

Species	Fitting indicator	Autocorrelation structure			
		**(3,3)**	(0,3)	(0,1)	(0,2)
D. dentex	Global deviance	1706.08	1717.0	1720.41	1753.18
	**AIC**	**1722.1**	1727.0	1728.4	1759.2
	SBC	1756.11	1748.27	1745.43	1771.94
		**(0,2)**	(0,3)	(1,0)	(0,1)
D. sargus	Global deviance	3486.84	3487.02	3492.58	3494.79
	**AIC**	**3494.84**	3497.02	3498.58	3500.79
	SBC	3511.86	3518.29	3511.34	3513.55
		**(0,2)**	(1,2)	(0,3)	(1,3)
D. vulgaris	Global deviance	5088.21	5087.29	5087.58	5087.2
	**AIC**	5096.21	5097.29	5097.58	5099.2
	SBC	5113.23	5118.56	5118.85	5124.72
		**(3,2)**	(3,1)	(2,3)	(2,1)
D. annularis	Global deviance	1745.15	1747.62	1757.23	1766.98
	**AIC**	**1759.15**	1759.62	1771.23	1776.98
	SBC	1788.92	1785.14	1801.01	1798.25
		**(3,2)**	(2,1)	(2,3)	(3,3)
D. cervinus	Global deviance	1297.27	1295.57	1296.28	1296.43
	**AIC**	**1307.27**	1309.57	1310.28	1312.43
	SBC	1328.54	1339.34	1340.06	1346.46
		**(2,2)**	(3,2)	(3,3)	(1,2)
D. puntazzo	Global deviance	1396.52	1396.23	1396.53	1402.63
	**AIC**	**1408.52**	1410.23	1412.53	1412.63
	SBC	1434.04	1440.01	1446.56	1433.9

Table S3. In this study we only used 78% of the total number of photos that were collected during the three years' study. The number of photos used for each month is indicated in table S1. The main reasons that contributed to the unavailability of the photos are: (*i*) problems of acquisition (this is a random error that sometimes occurred and the photo is not correctly acquired); (*ii*) cleaning of the camera's glass (a routine operation that was scheduled at least every month to guarantee sufficient visibility). Any photos taken on the day cleaning operations were performed were eliminated for possible disturbance of fish behavior by the underwater operators; (*iii*) technical problems of the whole observatory (e.g., during August 2012 the underwater camera did not work properly); (*iv*) winter storms during which the turbidity of the water was too high to allow fish counts; (*v*) external factors such as the theft of the connection copper cable which occurred in December 2014. In the table the number of photos used for each month during the three year of study is reported.

**Months**	**Years**		
	**2012**	**2013**	**2014**
January	1132	960	1461
February	1231	1274	1228
March	1440	1398	1386
April	1440	1358	1380
May	1438	1363	1396
June	1217	1241	1365
July	484	1376	1301
August	0	1430	1259
September	174	1308	1166
October	844	1427	1154
November	1367	1357	569
December	1215	1419	362

TableS4. All the possible model combination used manipulating presence (+) and absence (-) of independent variables.

	1	2	3	4	5	6	7	8
Temperature	+	-	+	+	+	-	-	-
Photoperiod	+	+	-	+	-	+	-	-
Salinity	+	+	+	-	-	-	+	-

Fig. S1. The frequency distribution of the daily count data of each of the 6 species

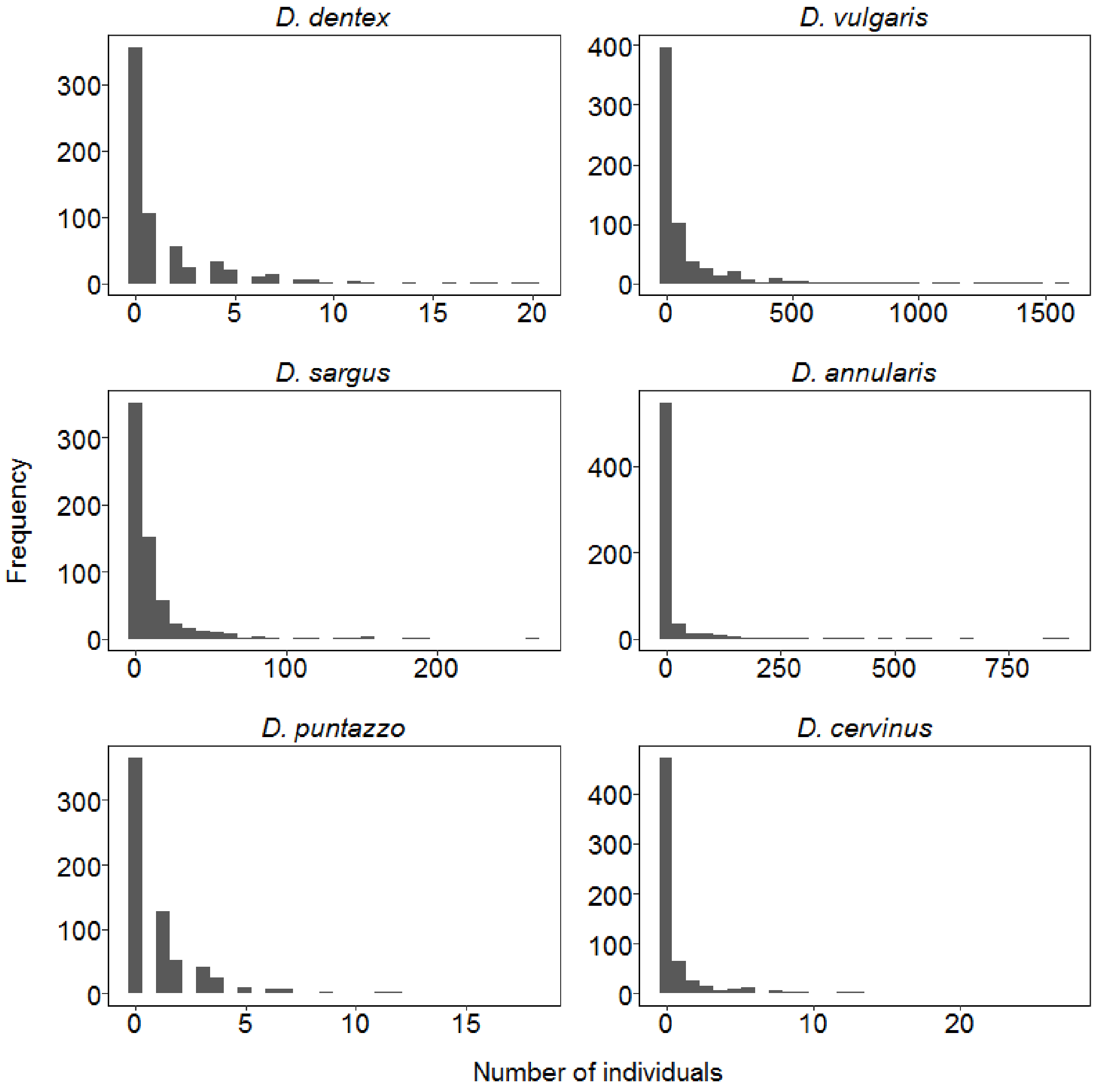

Fig S2. A representative (*D. dentex*) example of autocorrelation function plots (ACF) used to estimate the presence of autocorrelation in the residuals. The two plots represent a model without autocorrelation residual structure (a) and the best model selected (b; see also Table S2) with autocorrelation residual structure (e.g. *D. dentex*, GARMA (3,3)). ci: confidence intervals of 95%.

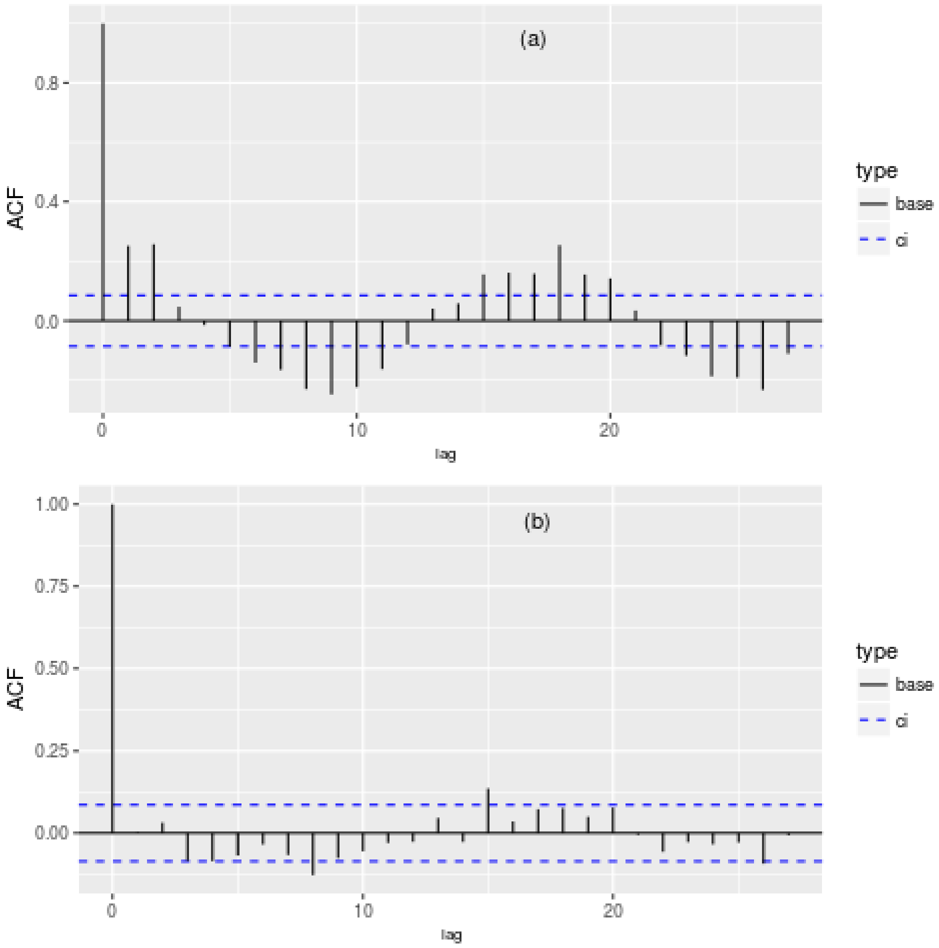

Fig S3. Boxplot of the daily number of individuals counted at the artificial reef according to the month (1-12) of the year for the six species studied here. The points represent the daily presence (number of individuals counted) of the species at the artificial reef during the 12 months of the three different years. In red the species that indicated a significant effect of the months as smoothing parameters (see also Table 1).

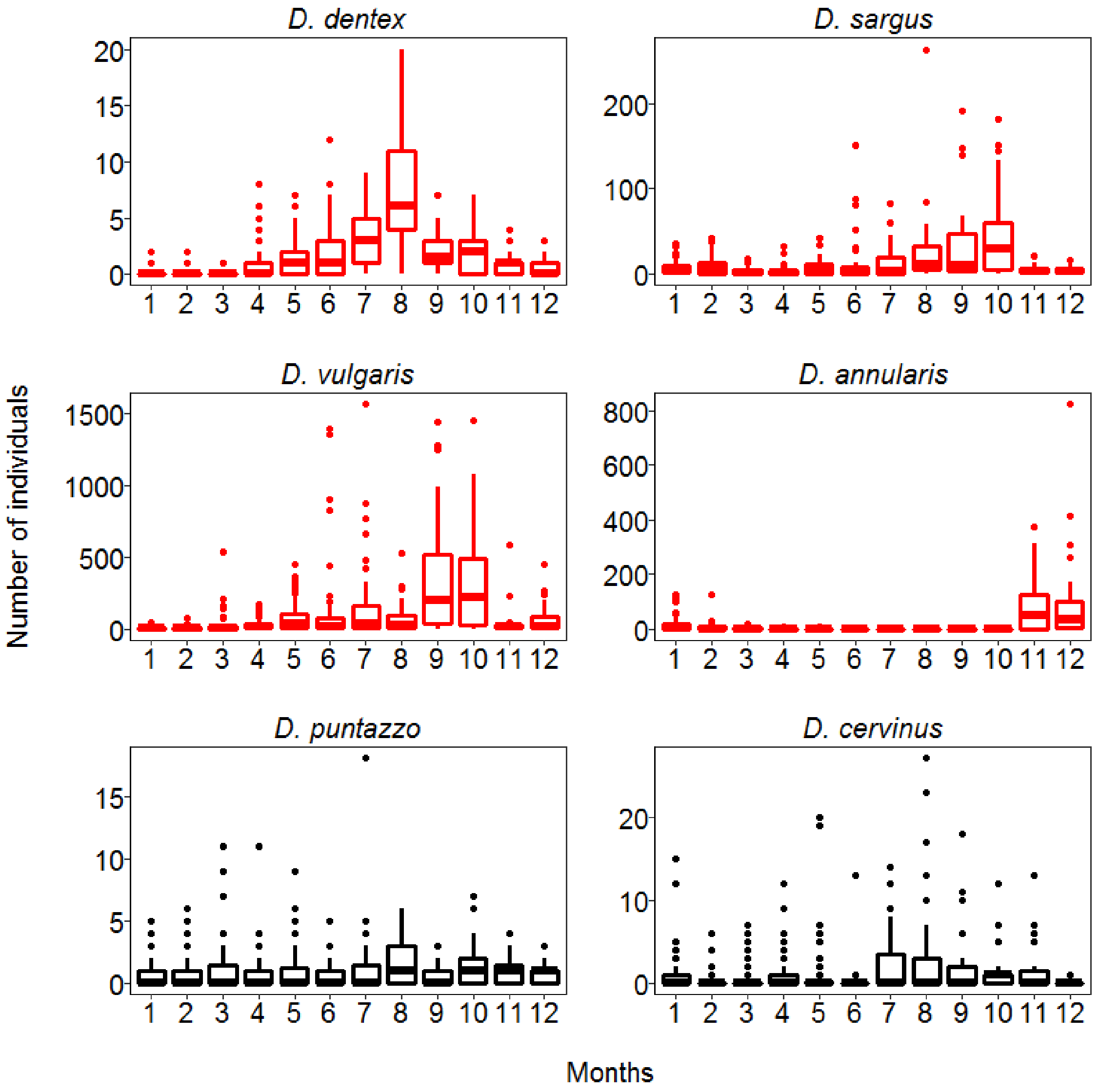

Supplement: Supplementary file 1 — Supplementary info [file 41598_2018_37954_MOESM1_ESM.pdf]
